# Long noncoding RNA CPS1-IT1 suppresses the metastasis of hepatocellular carcinoma by regulating HIF-1α activity and inhibiting epithelial-mesenchymal transition

**DOI:** 10.18632/oncotarget.9635

**Published:** 2016-05-26

**Authors:** Tong-Hong Wang, Cheng-Chia Yu, Yong-Shiang Lin, Tse-Ching Chen, Chau-Ting Yeh, Kung-Hao Liang, Tzong-Ming Shieh, Chi-Yuan Chen, Chuen Hsueh

**Affiliations:** ^1^ Tissue Bank, Chang Gung Memorial Hospital, Tao-Yuan, Taiwan; ^2^ Research Center for Industry of Human Ecology, Chang Gung University of Science and Technology, Tao-Yuan, Taiwan; ^3^ Graduate Institute of Health Industry Technology, Chang Gung University of Science and Technology, Tao-Yuan, Taiwan; ^4^ School of Dentistry, Chung Shan Medical University, Taichung, Taiwan; ^5^ Department of Dentistry, Chung Shan Medical University Hospital, Taichung, Taiwan; ^6^ Institute of Oral Sciences, Chung Shan Medical University, Taichung, Taiwan; ^7^ Department of Anatomic Pathology, Chang Gung Memorial Hospital, Chang Gung University School of Medicine, Tao-Yuan, Taiwan; ^8^ Liver Research Center, Department of Hepato-Gastroenterology, Chang Gung Memorial Hospital, Tao-Yuan, Taiwan; ^9^ Department of Dental Hygiene, College of Health Care, China Medical University, Taichung, Taiwan

**Keywords:** long noncoding RNA (lncRNA), hepatocellular carcinoma (HCC), metastasis

## Abstract

Recently, increasing numbers of long noncoding RNAs (lncRNAs), with both oncogenic and tumor-suppressive potential, have been found to be aberrantly expressed in various human cancers. However, the function of lncRNAs in hepatocellular carcinoma (HCC) progression remains largely unknown. In this study, we performed a comprehensive microarray analysis of lncRNA expression using human HCC specimens. After validation in 119 human HCC tissues, we identified a novel tumor suppressor lncRNA, CPS1 intronic transcript 1 (CPS1-IT1). To elucidate the clinical significance of CPS1-IT1 in HCC, correlations between CPS1-IT1 levels, clinical parameters, and survival outcomes were analyzed. *In vitro* and *in vivo* functional assays were also performed to dissect the potential underlying mechanisms. Expression of CPS1-IT1 was significantly decreased in 73% of HCC tissues, and patients with low CPS1-IT1 expression had poor survival outcomes. Furthermore, *in vitro* functional assays indicated that CPS1-IT1 significantly reduced cell proliferation, migration and invasion capacities through reduced Hsp90 binding to and activation of HIF-1α, thereby suppressing the epithelial-mesenchymal transition (EMT). An *in vivo* animal model also demonstrated the tumor suppressor role of CPS1- IT1 via decreased tumor growth and metastasis. In conclusion, lncRNA CPS1-IT1 acts as a tumor suppressor in HCC by reducing HIF-1α activation and suppressing EMT. The findings of this study establish a function for CPS1-IT1 in HCC progression and suggest its potential as a new prognostic biomarker and target for HCC therapy.

## INTRODUCTION

Human hepatocellular carcinoma (HCC) is the most common cancer of the liver and ranks third in cancer-related death worldwide [1]. According to the annual report of the Department of Health in Taiwan, HCC is also the second-most life-threatening malignancy for both sexes in Taiwan [2–4]. Currently, surgical resection, percutaneous ethanol injection and liver transplantation are the major therapeutic approaches for HCC. For patients who are not eligible for resection or those with metastasis, chemotherapy is the primary treatment. However, due to tumor recurrence, metastasis, and poor response to chemotherapy and radiotherapy, the therapeutic response is not satisfactory [5–7]; indeed, the recurrence rate during long-term follow-up is still more than 75% for patients with resectable HCC [8, 9]. Thus, it is important to explore new diagnostic and therapeutic molecular targets for HCC.

Previous research into mechanisms underlying tumorigenesis has mainly focused on protein-coding genes. However, recent studies have indicated that non-coding RNAs (ncRNAs) are also involved in regulating various physiologic functions, including tumor development, cell proliferation and migration, and apoptosis [10–12]. For instance, microRNAs (miRNAs), a well-known group of non-coding RNAs, mediate post-transcriptional gene silencing by inducing mRNA degradation or translational inhibition. It is estimated that approximately 30% of human genes are regulated by miRNAs, and miRNA deregulation is associated with several types of cancer [11, 13–16]. In addition to miRNAs, mounting evidence has indicated that long noncoding RNAs (lncRNAs), which are ncRNAs greater than 200 nt, are often deregulated in a wide variety of diseases, including cancer, Alzheimer's disease, and heart disease [17–21]. For example, the lncRNA HOTAIR has been shown to be overexpressed in HCC as well as breast, pancreatic and colon cancers, and HOTAIR can serve as a tumor biomarker [22]. The lncRNA MALAT1, which is overexpressed in esophageal squamous cell carcinoma (ESCC) and glioma, promotes tumor proliferation and metastasis capacity [23, 24], and the lncRNA Sox2ot promotes hepatocellular carcinoma cell metastasis and is correlated with a poor prognosis [25].

Recent studies have estimated that the number of lncRNAs is approximately 15,000, and most lncRNAs exhibit a tissue-specific pattern. Growing evidence has also revealed that lncRNAs regulate gene expression at different levels, including chromatin reprogramming, transcriptional processing, microRNA sponging and other processes. Thus, investigating the roles of lncRNAs in HCC may help to further understand HCC carcinogenesis.

Although lncRNAs have been found to be involved in various cellular processes, the functions of lncRNAs in HCC remain largely unclear. In this study, we identified a novel tumor suppressor lncRNA, CPS1 intronic transcript 1 (CPS1-IT1), by microarray analysis and subsequently validated this finding in HCC tissue specimens. We found expression of CPS1-IT1 to be significantly reduced in HCC tissues, and patients with low CPS1-IT1 expression had poor survival outcomes. Moreover, CPS1-IT1 could serve as an independent predictor for overall survival and disease-free survival in HCC. Further *in vitro* and *in vivo* experiments demonstrated the tumor suppressor role of CPS1-IT1: it reduced tumor growth and metastasis by decreasing Hsp90 binding to and activation of HIF-1α, thereby inhibiting the epithelial-mesenchymal transition. Thus, the findings of this study establish the function of CPS1-IT1 in HCC progression and suggest its potential as a new prognostic biomarker and target in HCC therapy.

## RESULTS

### Identification of lncRNAs deregulated in HCC tissues by microarray analysis

To screen lncRNAs involved in HCC progression, tumor and non-cancerous normal tissues obtained from three HCC patients were selected for microarray analysis (Affymetrix GeneChip^®^ Human Gene 2.0). We found 84 lncRNAs showing a > 2-fold change in tumor tissues compared to normal tissues (41 upregulated and 43 downregulated).

To validate these microarray findings, we randomly selected 10 lncRNAs with a fold change > 4 and validated their expression using quantitative real-time PCR in 15 paired HCC and normal liver samples. We found that the expression of only 5 of 10 lncRNAs was consistent with the microarray results. Among these lncRNAs, we further examined the role of CPS1-IT1 in hepatomagenesis.

### CPS1-IT1 expression is decreased in hepatocellular carcinoma

**Figure 1:**
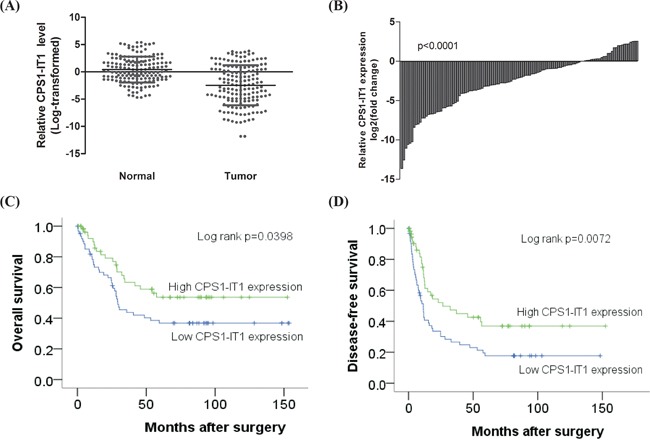
Relative expression of CPS1-IT1 in human hepatocellular carcinoma tissues **A.** Relative expression of CPS1-IT1 in 119 HCC tissues compared with corresponding non-cancerous normal tissues. CPS1-IT1 levels were examined using quantitative RT-PCR and normalized against GAPDH expression. **B.** Results are presented as the fold-change in tumor tissues relative to normal tissues. **C.** and **D.** Kaplan–Meier analysis of disease-free survival or overall survival according to CPS1-IT1 expression levels was performed. Low CPS1-IT1 expression (relative expression level < 2.65) in tumor tissues was associated with a poor prognostic outcome.

To elucidate the biological significance of the lncRNA CPS1-IT1 in HCC, we first examined CPS1-IT1 expression in 119 paired HCC and noncancerous normal liver tissues using quantitative real-time PCR. GAPDH was used as an internal control. The results showed that CPS1-IT1 was reduced in 73% (87/119, Figure 1A, 1B) of HCC tissues compared to normal liver tissues, suggesting a potential role as a tumor suppressor.

### Decreased CPS1-IT1 expression is associated with poor prognosis of HCC

To determine the clinical significance of CPS1-IT1, we further evaluated the correlation between CPS1-IT1 expression and survival outcome in 119 HCC patients. The clinical parameters, including patients' gender, age, carcinogenetic risk factors (HBV or HCV carrier, smoking, and alcoholism), cancer aggressiveness (degree of vascular invasion and capsule invasion), histology grading, tumor size, and serological markers (alpha-fetoprotein (AFP), albumin (Alb), bilirubin, creatinine (Cr), aspartate aminotransferase (AST), and alanine aminotransferase (ALT)) of the recruited HCC cohort are summarized in Table 1. The patients were first subclassified into high (ratio > 2.65) and low (ratio < 2.65) CPS1-IT1 expression groups, and the data were subjected to Kaplan-Meier survival analysis and a log rank test. The results showed that among the HCC cohort, lower CPS1-IT1 expression in normal tissues resulted in poor disease-free survival and overall survival (Figure 1C–1D). However, higher serological Alb and capsule invasion were significantly associated with better disease-free survival and overall survival (Table 2).

**Table 1:** Clinical parameters of the HCC patients included in this study

Clinical parameters	CPS1-IT1 < 2.65	CPS1-IT1 > 2.65
	N(%)	N(%)
Total number of patients	62(52.1)	57(47.9)
Gender		
Male	53(85.5)	48(84.2)
Female	9(14.5)	9(15.8)
Age		
< 55	25(40.3)	20(35.1)
> 55	37(59.7)	37(64.9)
Smoking		
Negative	35(58.3)	36(64.3)
Positive	25(41.7)	20(35.7)
Alcoholism		
Negative	39(66.1)	39(76.5)
Positive	20(33.9)	12(23.5)
HBV		
Negative	3(4.8)	2(3.6)
Positive	59(95.2)	54(96.4)
HCV		
Negative	2(3.2)	1(1.8)
Positive	60(96.8)	56(98.3)
Bilirubin		
< 0.9	31(50.0)	32(56.1)
> 0.9	31(50.0)	25(43.9)
AST		
< 52	41(67.2)	40(72.7)
> 52	20(32.8)	15(27.3)
ALT		
< 111	52 (86.7)	46(86.8)
> 111	8(13.3)	7(13.2)
Alb		
< 4	28(45.2)	23(40.4)
> 4	34(54.8)	34(59.7)
Cr		
< 1	22(36.7)	18(32.1)
> 1	38(63.3)	38(67.9)
AFP		
< 10	17(27.9)	18(33.3)
> 10	44(72.1)	36(66.7)
Tumor size		
< 3	22(35.5)	15(26.3)
> 3	40(64.5)	42(73.7)
Capsule invasion		
Absence	6(9.7)	7(12.3)
Presence	56(90.3)	50(87.7)
Vessel invasion		
Absence	25(40.3)	24(42.1)
Presence	37(59.7)	33(57.9)
Stage		
< 2	43(69.4)	38(66.7)
> 2	19(30.7)	19(33.3)

**Table 2:** Associations between CPS1-IT1 expression, clinical parameters and disease-free survival/overall survival

Clinical parameters	N	Disease-free survival (months)				Overall survival (months)			
		Mean	95%CI		p-value^#^	Mean	95%CI		p-value^#^
CPS1-IT1 expression									
< 2.65	62	25.91	17.17	34.65	0.007*	47.62	36.12	59.13	0.040*
> 2.65	57	33.62	23.43	43.80		46.68	36.41	56.96	
Gender									
Male	101	28.88	21.74	36.024	0.087	46.24	38.13	54.35	0.165
Female	18	33.63	14.47	52.789		52.40	28.32	76.49	
Age									
< 55	45	29.14	17.59	40.686	0.227	53.25	39.73	66.77	0.964
> 55	74	29.88	21.68	38.081		43.48	34.19	52.77	
Smoking									
Negative	71	27.86	20.22	35.501	0.112	43.63	34.85	52.41	0.426
Positive	45	29.96	17.60	42.314		51.23	36.48	65.99	
Alcoholism									
Negative	78	29.55	21.65	37.453	0.126	48.19	38.70	57.69	0.075
Positive	32	33.18	17.81	48.554		47.20	30.54	63.85	
HBV									
Negative	5	22.49	−16.84	61.811	0.555	40.09	−16.71	96.89	0.492
Positive	113	30.10	23.24	36.964		47.65	39.77	55.53	
HCV									
Negative	3	29.38	−76.81	135.576	0.841	58.72	−74.69	192.13	0.621
Positive	116	29.61	22.90	36.310		46.87	39.15	54.60	
Bilirubin									
< 0.9	63	26.65	18.36	34.927	0.414	41.91	32.01	51.81	0.649
> 0.9	56	32.93	22.20	43.653		53.10	41.14	65.06	
AST									
< 52	81	31.06	22.77	39.343	0.390	47.72	38.02	57.42	0.567
> 52	35	27.46	15.23	39.687		47.97	34.37	61.57	
ALT									
< 111	98	30.55	22.87	38.219	0.308	46.80	37.98	55.61	0.989
> 111	15	24.61	8.97	40.244		52.89	33.25	72.54	
Alb									
< 4	51	22.42	13.13	31.707	0.036*	35.65	25.61	45.70	0.016*
> 4	68	34.99	25.76	44.213		55.81	45.00	66.62	
Cr									
< 1	40	26.02	14.64	37.396	0.702	45.85	31.87	59.83	0.715
> 1	76	30.34	21.96	38.721		46.75	37.17	56.33	
AFP									
< 10	35	36.00	21.82	50.188	0.154	50.17	34.60	65.73	0.160
> 10	80	27.80	20.08	35.525		47.25	38.03	56.48	
Tumor size									
< 3	37	36.15	22.54	49.767	0.315	55.43	40.11	70.74	0.214
> 3	82	26.64	19.15	34.135		43.45	34.66	52.24	
Capsule invasion									
Absence	13	10.39	1.80	18.975	0.050*	21.89	6.68	37.10	0.031*
Presence	106	31.96	24.71	39.203		50.27	42.03	58.51	
Vessel invasion									
Absence	49	33.85	23.83	43.872	0.233	47.20	36.22	58.18	0.806
Presence	70	26.62	17.71	35.544		47.16	36.45	57.86	
Stage									
< 2	81	29.66	21.31	38.019	0.589	48.41	38.68	58.14	0.301
> 2	38	29.47	18.37	40.569		44.53	31.98	57.07	

# Log rank test.

To determine potential independent predictors for postoperative survival, a stepwise multivariate Cox proportional hazard model was employed. Higher CPS1-IT1 and Alb expression in normal tissues was associated with a reduced lethal risk of 0.55× and 0.64×, respectively. However, for overall survival, only high CPS1-IT1 expression reduced the lethal risk by 0.57× (Table 3).

**Table 3:** Stepwise multivariate Cox proportional hazard model for independent predictors of postoperative survival

Factors	Multivariate			
	HR	95%CI		p-value
Disease-free survival				
High CPS1-IT1 (> 2.65 vs. < 2.65)	0.55	0.34	0.87	0.011*
Alb (> 4 vs. < 4)	0.64	0.41	1.01	0.053
Overall survival				
High CPS1-IT1 (> 2.65 vs. < 2.65)	0.57	0.34	0.98	0.042*

### CPS1-IT1 acts as a tumor suppressor by suppressing cell proliferation and migration *in vitro*

**Figure 2:**
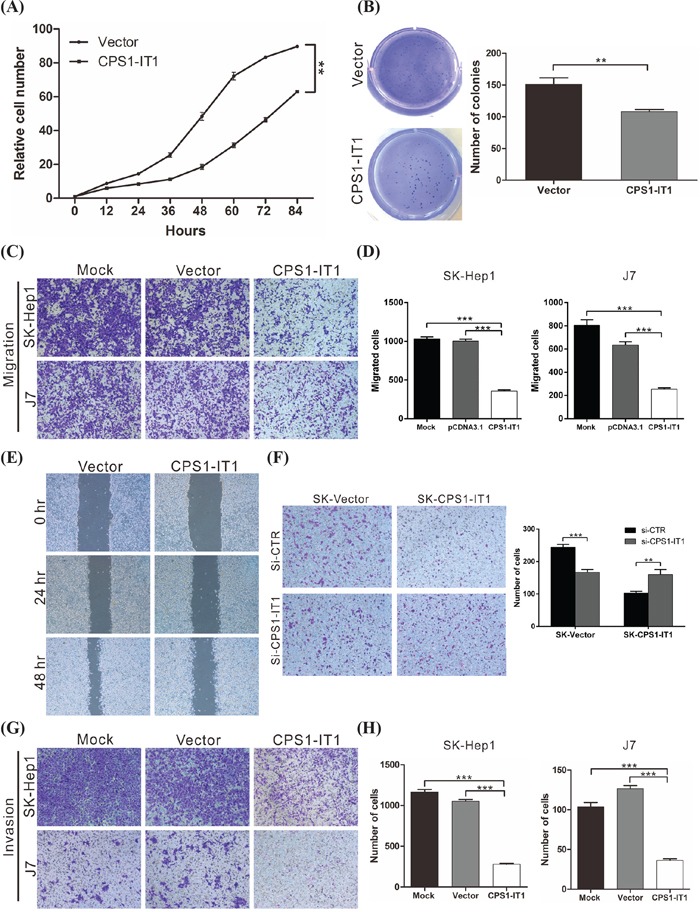
CPS1-IT1 suppresses cell proliferation, migration and invasion *in vitro* **A.** The proliferation capacity of cells was monitored at the indicated time points using an xCELLigence real-time cell analyzer. Forced expression of CPS1-IT1 significantly reduced SK-Hep1 cell growth compared to the vector control at 84 hours. p < 0.01 (**). **B.** Colony formation ability was analyzed in cells at 12 days after transfection with the CPS1-IT1 plasmid or empty vector. CPS1-IT1 significantly inhibited the colony formation ability of SK-Hep1 cells. **C.** Cell migration was compared between J7 and SK-Hep1 cells transfected with either the CPS1-IT1 expression plasmid or empty vector. Overexpression of CPS1-IT1 significantly reduced cell migration ability. Quantification of the cell migration assays is presented in **D.** p < 0.001 (***). **E.** Results of the wound-healing assay were compared between SK-Hep1 cells transfected with the CPS1-IT1 plasmid or empty vector. Overexpression of CPS1-IT1 inhibited the wound-healing ability of SK-Hep1 cells. **F.** Cell migration was compared between SK-Hep1 cells overexpressing CPS1-IT1 or with silenced CPS1-IT1 expression. Overexpression of CPS1-IT1 significantly reduced the cell migratory ability, whereas silencing of CPS1-IT1 enhanced the cell migratory capacity (left panel). Quantification of the cell migration assays is shown in the right panel. p < 0.001 (***). **G.** Invasion assays were performed with SK-Hep-1 and J7 cells on Matrigel-coated polyethylene terephthalate membrane inserts. Five different 200× fields were imaged to quantify migrating or invading cells. Quantification of the invasive cells is shown in **H.**

To determine the biological functions of CPS1-IT1 during tumor progression, CPS1-IT1 was overexpressed in J7 and SK-Hep1 cell lines, and cell proliferation was monitored using an xCELLigence real-time cell analyzer. We found that forced CPS1-IT1 expression significantly reduced proliferation of the SK-Hep1 cell line, inhibiting proliferation capacity by approximately 29.7% at 84 hours compared to cells transfected with the empty vector (p < 0.01**) (Figure 2A). Similarly, colony-formation ability was reduced by CPS1-IT1 in both HCC cell lines (Figure 2B). Furthermore, we examined cell migration capacity using transwell and wound healing assays (Figure 2C–2E). The migration capacities of both CPS1-IT1-overexpressing cell lines were reduced compared to the control group, and the effects triggered by CPS1-IT1 were significantly rescued by treatment with CPS1-IT1 siRNA (Figure 2F). Similar results were observed in the cell invasion assay, with the invasion capacity of both CPS1-IT1-overexpressing cell lines being reduced by approximately 75% and 70%, respectively, compared to the control group (Figure 2G–2H). Taken together, these results suggested that CPS1-IT1 suppressed cancer cell proliferation as well as migration and invasion capacities.

### CPS1-IT1 is associated with Hsp90 and inhibits the epithelial-mesenchymal transition

**Figure 3:**
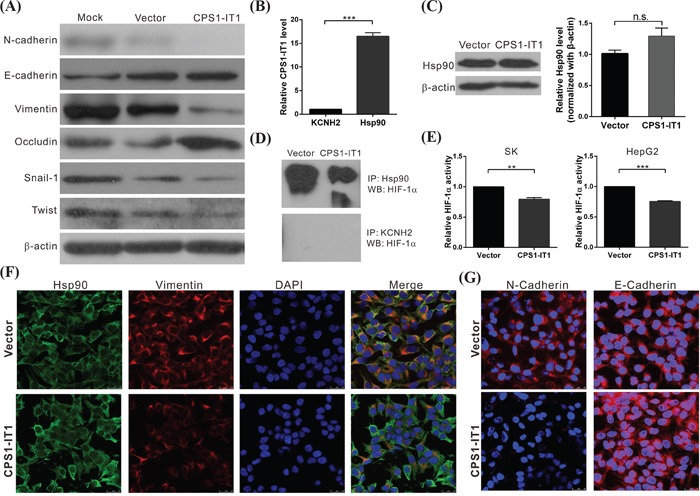
CPS1-IT1 associates with Hsp90 and inhibits the epithelial-mesenchymal transition **A.** Western blotting analysis of EMT-associated proteins after CPS1-IT1 plasmid or empty vector transfection. β-Actin served as the internal control. **B.** RIP experiments were performed on SK-Hep1 cell lysates using an antibody against Hsp90. The KCNH2 antibody served as a negative control. Purified RNA was subjected to qRT-PCR for CPS1-IT1 detection. The results revealed an association between CPS1-IT1 and Hsp90. **C.** Western blotting analyses revealed the levels of Hsp90 in SK-Hep1 cells transfected with the CPS1-IT1 plasmid or vector only. Overexpression of CPS1-IT1 did not affect the protein levels of Hsp90. **D.** Binding affinity between Hsp90 and HIF-1α was analyzed using a co-immunoprecipitation assay. CPS1-IT1 reduced the binding between Hsp90 and HIF-1α. **E.** HIF-1a activity was analyzed using a dual luciferase reporter assay. CPS1-IT1 reduced the activity of HIF-1α in SK-Hep1 and HepG2 cells. **F.** and **G.** Expression of Hsp90 and EMT-associated proteins in SK-Hep1 cells after CPS1-IT1 plasmid or empty vector transfection was examined using immunofluorescence staining. CPS1-IT1 reduced vimentin and N-cadherin expression but did not affect Hsp90 levels.

Occurrence of the epithelial-mesenchymal transition (EMT) during tumorigenesis may increase the motility and invasiveness of cancer cells, and malignant transformation may be associated with signaling pathways that promote EMT [26]. To further investigate associations between EMT and CPS1-IT1 in HCC, we analyzed the expression patterns of EMT-associated proteins, including E-cadherin, N-cadherin, occludin, vimentin, snail, and twist, in the SK-Hep1 cell line. Expression of the EMT-promoting proteins N-cadherin, vimentin, snail, and twist was reduced in CPS1-IT1-overexpressing cells, whereas the levels of E-cadherin and occludin were increased (Figure 3A). These results suggested that CPS1-IT1 exerted its tumor suppressive effects by inhibiting EMT.

Recent studies have indicated that lncRNAs can bind to proteins and alter their functions; these molecules are thus involved in regulating a variety of biological processes [27, 28]. To further characterize the potential mechanism of CPS1-IT1 in EMT inhibition, an RNA pull-down assay followed by LC-MASS analysis was performed. As shown in Table 4, several proteins, including heat shock protein 90 (Hsp90), heat shock protein 70 (Hsp70) and the neuroblast differentiation-associated protein AHNAK, were identified. To further validate associations between CPS1-IT1 and these proteins, an RNA immunoprecipitation (RIP) assay was performed using antibodies against these proteins. Significantly higher enrichment of CPS1-IT1 was observed with the anti-Hsp90 antibody compared with a non-specific IgG control KCNH2 antibody (Figure 3B). These results indicated a high level of association between CPS1-IT1 and Hsp90.

**Table 4:** Mass spectrometry analysis of the proteins pulled down by the lncRNA CPS1-IT1

Hits	Description	Protein score	Protein mass (Da)	PSMs	Peptides	Protein coverage %
1	Neuroblast differentiation-associated protein AHNAK	3013	629,213	74	43	11.3
2	Heat shock protein HSP 90-beta	2057	83,554	58	22	30.9
3	Heat shock cognate 71-kDa protein	1933	71,082	49	20	33.3
4	Heat shock protein HSP 90-alpha	1841	85,006	48	19	28.4
5	Filamin-A	1543	283,301	39	23	12.3
6	78-kDa Glucose-regulated protein	1502	72,402	37	19	34.3
7	60-kDa heat shock protein, mitochondrial	1500	61,187	34	15	35.3
8	Filamin-C	1491	293,407	35	19	9.8
9	Myosin-9	1428	227,646	30	17	10.8
10	Stress-70 protein, mitochondrial	1418	73,920	33	16	31.2

### CPS1-IT1 acts as a co-chaperone and alters Hsp90 and HIF-1α binding affinity

The protein chaperone Hsp90 is a major regulator of the activities of different transcription factors. It has been previously demonstrated that Hsp90 promotes EMT in colorectal cancer via activation of HIF-1α, which results in a subsequent downregulation of Hsp90, leading to inhibition of EMT, motility, and invasiveness [29, 30]. To further validate the regulation of Hsp90 by CPS1-IT1, we examined whether CPS1-IT1 affects the stability of Hsp90 using western blotting analysis. However, we did not observe any difference in Hsp90 levels between cells transfected with the CPS1-IT1 plasmid or empty vector (Figure 3C). The same results were also observed using an immunofluorescence staining assay. Although CPS1-IT overexpression reduced the expression of EMT-promoting proteins such as N-cadherin and vimentin, Hsp90 expression was not altered (Figure 3F–3G). Taken together, these results suggested that CPS1-IT1 did not regulate the stability of Hsp90.

To investigate whether CPS1-IT1 is involved in Hsp90-mediated HIF-1α activation, the binding affinity between Hsp90 and HIF-1α was analyzed using a co-immunoprecipitation assay. As shown in Figure 3D, HIF-1α co-immunoprecipitated with Hsp90; however, a significant reduction in the interaction between HIF-1α and Hsp90 was detected in cells overexpressing CPS1-IT1. Hsp90 levels on the same blot were also analyzed, demonstrating that a similar amount of Hsp90 was immunoprecipitated in each sample (data not shown). Furthermore, HIF-1α activity was examined, and we found reductions in CPS1-IT1-overexpressing cells compared to the control group (Figure 3E). These results indicated that CPS1-IT1 interacted with Hsp90 and reduced the binding affinity between Hsp90 and HIF-1α, thereby resulting in reduced HIF-1α activation. Inactivation of HIF-1α resulted in decreased expression of EMT-related proteins.

### CPS1-IT1 suppresses tumor growth and metastasis *in vivo*

**Figure 4:**
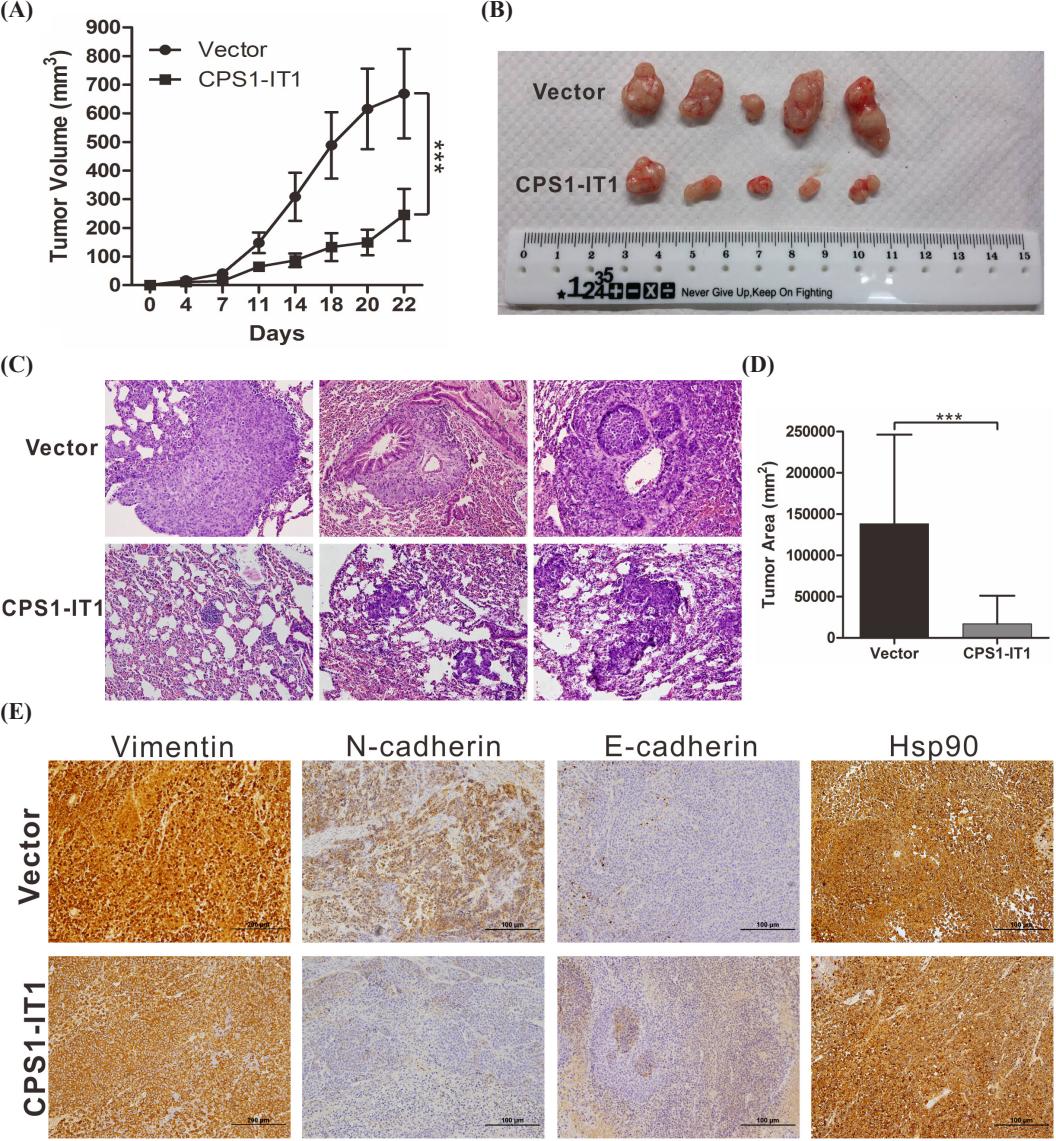
CPS1-IT1 reduces tumor growth and metastasis of HCC cells *in vivo* **A.** SK-Hep1 cells stably transfected with pCDNA3.1-CPS1-IT1 or empty vector were inoculated into nude mice (n = 5). Tumor volumes were calculated every 3 days after injection. Bars indicate S.D. **B.** Representative images showing the tumor xenografts at 4 weeks after implantation. CPS1-IT1 expression significantly reduced tumor growth. **C.** Representative images of lung metastasis as illustrated by H&E staining in nude mice at 6 weeks after tail vein injection with SK-Hep1 cells stably transfected with pCDNA3.1 or pCDNA3.1-CPS1-IT1. CPS1-IT1 significantly reduced tumor metastasis. Magnification: 200×. **D.** Histological analysis of lung tumor volume in the control and CPS1-IT1-overexpressing groups. The mean ± S.D. is shown. (n = 5). ** p < 0.01, *** p < 0.001. **E.** CPS1-IT1 reduced the expression of EMT-promoting proteins, as assessed using immunohistochemical staining.

To further validate the tumor suppressor role of CPS1-IT1 in HCC, *in vivo* xenograft and tail injection migration assays were performed. Consistent with previous results, the growth of tumors from CPS1-IT1 xenografts was significantly reduced compared with the control group at 21 days after injection (Figure 4A, 4B). An inhibitory effect of CPS1-IT1 on HCC lung metastasis was also observed (Figure 4C, 4D). Immunohistochemical staining was performed to confirm the regulation of EMT by CPS1-IT1. Similar to the results obtained in the *in vitro* cell assay, expression of EMT-promoting proteins, such as vimentin and N-cadherin, was decreased in CPS1-IT1-overexpressing tumors, whereas expression of E-cadherin was increased. No differences in the levels of Hsp90 between the CPS1-IT1-overexpressing and control groups were observed (Figure 4E).

## DISCUSSION

In addition to microRNAs, lncRNAs have emerged as important factors in cell biology. Evidence to date links lncRNA dysregulation to diverse human diseases, including cancers [31–34]. However, the function of lncRNAs in HCC progression remains largely unknown. In this study, we identified a novel tumor suppressor lncRNA: CPS1-IT1. Expression of CPS1-IT1 was significantly reduced in HCC tissues, and low CPS1-IT1 expression was correlated with poor outcomes. *In vitro* functional assays and an *in vivo* animal model demonstrated that CPS1-IT1 reduced cell proliferation as well as migration and invasion capacities, thereby suppressing EMT. Together, these findings indicated that CPS1-IT1 functions as a tumor suppressor in HCC and may serve as a prognostic marker for HCC.

Invasion and metastasis are the main causes of cancer-related mortality, and EMT is well known to increase the motility and invasiveness of cancer cells [35, 36]. In this study, we observed that CPS1-IT1 significantly reduced cell migration and invasion capacities. Furthermore, expression of EMT-promoting proteins, such as N-cadherin, vimentin, snail and twist, was significantly reduced in CPS1-IT1-overexpressing cells. These results indicated that CPS1-IT1 could decrease HCC invasion and metastasis by suppressing EMT. This finding was further supported by prognostic analysis. Decreased CPS1-IT1 expression was associated with shorter metastasis-free and overall survival after surgery in HCC patients, supporting the hypothesis that CPS1-IT1 can serve as a molecular prognostic factor that can be used to identify high-risk patients for further intense treatment.

The molecular mechanisms of lncRNAs are diverse. lncRNAs can function in the following ways: (i) as a decoy to locate transcription factors; (ii) as a regulatory signal for transcription; (iii) as a scaffold to bridge different proteins; (iv) as a ‘sponge’ to sequester microRNAs; (v) as a guide for proteins to reach their targets; and (vi) as a modified protein that can allosterically alter the functions of other proteins [37, 38]. For instance, the lncRNA CCND1 allosterically regulates the activation of TLS [39], and the lncRNA rncs-1 modulates the expression of Dicer-regulated genes [40]. In addition, sno-lncRNAs strongly associate with Fox family splicing regulators to alter splicing patterns. Similar to CCND1, we found that CPS1-IT1 interacts with Hsp90, a dimeric molecular chaperone that is required for the activation and stabilization of numerous proteins; many of these proteins are involved in essential cellular processes, such as signal transduction pathways [41, 42]. The activation process is regulated by large ATP-induced conformational changes, co-chaperones and posttranslational modifications. In eukaryotic cells, more than 20 co-chaperones have been identified as regulating the function of HspP90 in various ways, such as inhibition and activation of its ATPase activity as well as recruitment of specific client proteins. In addition to promoting EMT in colorectal cancer via activation of HIF-1α by Hsp90 [29], several reports have also demonstrated that Hsp90 promotes α-SMA and p-p38 expression and reduces that of E-cadherin in HCC cells, thereby promoting EMT. Consistent with these findings, we suggest that based on the EMT suppression effects of CPS1-IT, this lncRNA may act as an Hsp90 co-chaperone, thereby altering the conformation of Hsp90 and its binding affinity toward HIF-1α. According to our results, inactivation of HIF-1α resulted in decreased expression of EMT-associated proteins.

DNA methylation at gene promoters is crucial for gene silencing and may function as an epigenetic regulator of lncRNA expression. For instance, the lncRNA MEG3, the expression of which is indirectly regulated by mir-29a in HCC cells, inhibits the activity of DNA methyltransferase and reduces MEG3 expression [43, 44]. Thus, further studies on promoter hypermethylation are required to determine the upstream regulatory mechanisms of decreased CPS1-IT expression in HCC.

In conclusion, we identified a long noncoding RNA named CPS1-IT1 and established its tumor suppressor role in HCC carcinogenesis. To the best of our knowledge, this is the first study to address the function of CPS1-IT1 in HCC. Taken together, our results provide new insight into the role of lncRNAs in the development of HCC and support the notion that CPS1-IT1 may serve as a prognostic biomarker and a potential therapeutic molecular target for the treatment of HCC.

## MATERIALS AND METHODS

### Tissues

The human HCC and corresponding non-cancerous normal tissues used in this study were obtained from 119 HCC patients who underwent surgical resection at Lin-Kou Chang Gung Memorial Hospital between 2000 and 2012. Clinical and pathological characteristics were obtained from patient charts. Tumors were staged according to the seventh edition of the American Joint Committee on Cancer, and the histological grade was scored according to World Health Organization classification criteria. This study was approved by the Ethics Committee of Chang Gung Memorial Hospital, and written informed consent was obtained from each patient.

### Cell lines, antibodies, siRNAs and plasmid construction

The HCC cell lines J7, HepG2, and SK-Hep1 were cultured in DMEM medium containing 10% fetal bovine serum at 37°C in a 5% CO_2_ atmosphere. Polyclonal antibodies against Hsp90, HIF-1α, E-cadherin, N-cadherin, vimentin, occludin, twist, snail and β-actin were purchased from Genetex (Irvine, CA) and Cell Signaling Technology (Beverly, MA). Secondary antibodies were purchased from Santa Cruz Biotechnology (Santa Cruz, CA). All siRNAs were purchased from Applied Biosystems (Foster City, CA). pCDNA3.1-CPS1-IT1, a CMV-based expression and neomycin-selective plasmid containing lncRNA-CPS1-IT1, was constructed by GenScript Co. (Piscataway, NJ).

### Generation of stable cell lines

For the production of stable clones, cell lines were seeded in 10-cm dishes at a density of 1×10^6^ cells/well and grown overnight; the cells were then transfected with 3 μg of either pCDNA3.1-CPS1-IT1 or pCDNA3.1 plasmid using Lipofectamine 2000 (Invitrogen, Carlsbad, CA). Twenty-four hours after transfection, the medium was replaced with fresh medium containing 0.8 mg/ml G418, and the cells were cultured and selected in this cloning medium for 4 weeks. Well-separated colonies were harvested using cloning cylinders and trypsinization; the cells were transferred to 24-well plates, and the cultures were further expanded. Once actively growing, clone stocks were routinely maintained in culture medium supplemented with 0.8 mg/ml G418.

### Microarray analysis

Total RNA from tumor and non-cancerous normal tissues obtained from three HCC patients was isolated using the TRIzol reagent (Invitrogen, Carlsbad, CA), amplified and transcribed into cDNA. The biotinylated cDNA was hybridized to the Human Gene 2.0 ST Array (Affymetrix, Santa Clara, CA), as performed by Chang Gung Memorial Hospital Genomics Center (Taiwan), following the manufacturer's instructions. The microarray data were analyzed using Transcriptome Analysis Console (TAC) software version 3.0 (Affymetrix).

### Detection of the lncRNA CPS1-IT1 in HCCs using quantitative real-time RT-PCR

Total RNA from each tissue was isolated using an RNeasy mini kit (QIAGEN, Gaithersburg, MD, USA), followed by treatment with RQ1 RNase-free DNase (Promega, Madison, WI) according to the manufacturer's instructions. Two micrograms of treated RNA sample was subjected to reverse transcription (RT). The RT product was subjected to quantitative real time RT-PCR for detection of lncRNA CPS1-IT1 expression using the TaqMan non-coding RNA expression assay (Applied Biosystems, Foster City, CA); GAPDH was used as an internal control.

### Transfection and western blotting analysis

Cell lines were seeded overnight in 6-well plates at a density of 3×10^5^ cells/well. The cells were transfected with 1 μg of either pCDNA3.1-CPS1-IT1 or pCDNA3.1 plasmid using Lipofectamine 2000 (Invitrogen, Carlsbad, CA) according to the manufacturer's instructions. Forty-eight hours later, the transfected cells were washed twice with PBS and lysed in 200 μl of RIPA lysis buffer (50 mM Tris–HCl, pH 7.4, 150 mM NaCl, 1 mM EDTA, 1% Triton X-100, 1% sodium deoxycholate, 0.1% SDS) containing protease inhibitors. Proteins (100 μg) from the supernatant were loaded onto an SDS polyacrylamide gel, following by western blotting analysis to detect the levels of E-cadherin, N-cadherin, occludin, vimentin, twist, snail and β-actin. The immunoreactive bands were visualized using an ECL system (NEN Life Science Products, Boston, MA) and developed using x-ray films. The amount of protein in each band was quantified using ImageQuant 5.2 (GE Healthcare, Piscataway, NJ).

### Immunofluorescence staining

Immunofluorescence staining was performed as previously described [45]. Briefly, cells seeded on a 4-well chamber slide (Merck Millipore, Darmstadt, Germany) at a density of 1×10^4^ cells/well were transfected with 1 μg of either pCDNA3.1-CPS1-IT1 or pCDNA3.1 plasmid. Forty-eight hours after transfection, the cells were harvested and fixed in ice-cold acetone for 10 min. The slides were washed 3 times with 1× PBS, blocked with 5% goat serum, and incubated with antibodies against Hsp90, vimentin, N-cadherin and E-cadherin (Cell Signaling Technology). Next, the slides were labeled with Alexa Flour 488-conjugated goat anti-rabbit IgG and Alexa Fluor 546-conjugated goat anti-mouse IgG (Invitrogen) and counter-stained with DAPI. The cells were then analyzed using confocal fluorescence microscopy.

### Cell proliferation assay

Cell proliferation capacity was monitored using an xCELLigence real-time cell analyzer (Roche Life Science, Indiana, USA) and examined by a colony formation assay according to the manufacturer's instructions. For the colony formation assay, cells transfected with a plasmid expressing CPS1-IT1 or the empty vector were seeded onto 6-well plates at a density of 500 cells/well and maintained for 12 days in DMEM containing 10% FBS. The medium was replaced every 3 days. Colonies were fixed with methanol twelve days later and stained with 0.1% crystal violet (Sigma–Aldrich, St. Louis, MO, USA). Visible colonies were imaged and manually quantified.

### Cell migration and invasion assays

Cell migration activity was analyzed using a wound-healing assay and a transwell migration assay as previously described [12]. For the wound-healing assay, treated SK-Hep1 cells were plated onto 6-well plates and cultured to 90% confluence. Cells were scraped with a p200 tip (time 0), and the medium was replaced with low-serum culture medium. The migration distances of the cells were measured from images (five fields) taken at the indicated time points.

The migration and invasion abilities of J7 and SK-Hep1 cells were assessed using ThinCert Tissue Cell Culture Inserts (Greiner Bio-One, Kremsmunster, Austria) containing an 8-μm mean pore size membrane. For the migration assay, the cells were trypsinized and suspended in serum-free culture medium (DMEM) to a final concentration of 5×10^5^ cells/ml. The lower chambers were filled with 500 μl of complete medium (DMEM supplemented with 10% FBS), and 100-μl aliquots of the cells were loaded into each upper well. The chambers were incubated in a humidified 5% CO_2_ incubator at 37°C for 24 hours. The cells were fixed with 500 μl of methanol for 15 minutes; to remove the non-migrating cells, cells on the inner surface of the upper chambers were wiped using cotton swabs. The membrane was washed with 500 μl of PBS and stained with 500 μl of crystal violet for 20 minutes at room temperature. After the cells were washed with 500 μl of PBS, the stained cells were imaged using ImagePro 6.2 software. Counts were obtained from five random fields at 100× magnification. For the invasion assay, the membrane was coated with 30 mg/cm^2^ Matrigel (ECM gel, Sigma–Aldrich, St. Louis, MO) to form a matrix barrier. The procedure for the migration assay was the same as described above, except that the migration time was 48 hours.

### *In vitro* transcription and RNA pull-down assay

*In vitro* transcription was performed as previously described [46]. Briefly, the pCDNA3.1-CPS1-IT1 plasmid containing a T7 promoter was linearized by BamHI digestion, purified, and used as a template for *in vitro* transcription. The template was incubated in ATP, CTP, GTP, and UTP (1 mM each) plus T7 RNA polymerase in 1× transcription buffer. The *in vitro*-transcribed RNA was purified using an RNeasy purification kit (Qiagen, Germany), and the transcripts were labeled with biotin using a Thermo Scientific Pierce RNA 3′ Desthiobiotinylation Kit (Thermo Fisher Scientific, MA, USA). The RNA-protein binding reaction was performed using freshly harvested SK-Hep1 cells and a Pierce^™^ Magnetic RNA-Protein Pull-Down Kit (Thermo Fisher Scientific, MA, USA). The reaction products were then subjected to mass spectrometry for protein identification.

### Protein identification via mass spectrometry

After protein separation by SDS-PAGE, the gel was stained with EZBlue (Sigma–Aldrich, St. Louis, MO, USA). The detected bands, in addition to the control region in which no proteins were detected, were excised. In-gel tryptic digestion was performed according to the manufacturer's protocol. The tryptic digest was then analyzed by mass spectrometry. Matrix-assisted laser desorption ionization–time of flight mass spectrometry was initially performed to identify peptide mass fingerprints. Specific peaks were selected for inclusion in the tandem mass spectrometry analysis to identify the peptide sequences. Proteins were identified using MASCOT software.

### RNA immunoprecipitation assay

Protein-RNA complexes were immunoprecipitated using 3 μg of an anti-Hsp90 antibody, and RNA was purified using an RNeasy extraction kit (Qiagen, Hilden, Germany). The anti-KCNH2 antibody was used as a negative control. To detect CPS1-IT1 expression, the extracted RNA was subjected to quantitative real-time RT-PCR using a TaqMan non-coding RNA assay (Applied Biosystems, Foster City, CA, USA).

### HIF-1α activity assay

HIF-1α activity was analyzed using a Cignal HIF Reporter assay (Qiagen, Hilden, Germany). Cell lines were seeded in 6-well plates at a density of 3×10^5^ cells/well overnight. The cells were transfected with 1 μg of either pCDNA3.1-CPS1-IT1 or pCDNA3.1 plasmid. Twenty-four hours after transfection, the cells were transfected with 1 μg HIF-1 reporter mixture. Another 48 hours later, the cells were harvested and subjected to a luciferase assay.

### Mice

Male BALB/C nude mice that were 6-8 weeks old (purchased from the National Laboratory Animal Center, Taipei, Taiwan) were housed under pathogen-free conditions with a 12-hour light/12-hour dark schedule and provided autoclaved standard chow and water. The mice were bred at the Animal Center of Chang-Gung Hospital (Taoyuan, Taiwan) according to the Guidelines for the Care and Use of Laboratory Animals (NIH). All experiments related to the animal studies were approved by the Institutional Animal Care and Use Committee (IACUC) at Chang-Gung Hospital.

### Tumorigenicity and *in vivo* metastasis assays

A total of 1×10^6^ cells of the J7 and SK-Hep1 cell lines, stably expressing the CPS1-IT1 or pCDNA3.1 plasmid, were resuspended in 100 μl of saline with 50% Matrigel (BD Biosciences) and implanted subcutaneously into the left and right flank regions of the mice. Tumor volume growth was recorded weekly using digital calipers. For the *in vivo* metastasis assay, the same number of transfected cells was injected into the tail vein of the nude mice, which were sacrificed at 6 weeks. Lung metastasis was examined using H&E staining with a microscope, and the tumor volume was measured in five random fields at 100× magnification.

### Immunohistochemistry

Tissues were fixed in formalin and embedded in paraffin, and 2-μm-thick consecutive sections were cut and mounted onto glass slides. The slides were first incubated at 65°C for 1 hour and then deparaffinized in xylene, rehydrated in graded ethanol solutions, and finally boiled in Trilogy reagent (Cell Marque, Rocklin, CA) for 10 minutes for antigen retrieval. After washing with 1× phosphate-buffered saline (PBS), the slides were immersed in 3% hydrogen peroxide for 10 minutes to suppress endogenous peroxidase activity. After three rinses with 1× PBS, the sections were subsequently incubated with antibodies against Hsp90, vimentin, N-cadherin and E-cadherin for 1 hour at room temperature. After three rinses with 1× PBS, the slides were incubated with a biotinylated secondary antibody (Dako, Glostrup, Denmark) for 25 minutes. Following three rinses with 1× PBS, horseradish-peroxidase conjugated streptavidin was added for 25 minutes at room temperature. Peroxidase activity was detected using 3,3-diaminobenzidine (DAB) (Dako) at room temperature. The slides were then counterstained with hematoxylin.

### Statistical analysis

The original real-time PCR data and data for the western blotting and migration assay analyses were recorded as continuous variants and analyzed using Student's *t*-test. All statistical analyses were performed using SPSS 16.0 and Excel 2007. All statistical tests were two-sided, and p-values < 0.05 (*), < 0.01 (**), or < 0.001 (***) were considered to be significant.
